# Arp2/3-mediated bidirectional actin assembly by SPIN90 dimers

**DOI:** 10.1038/s41594-025-01665-8

**Published:** 2025-09-15

**Authors:** Tianyang Liu, Luyan Cao, Miroslav Mladenov, Guillaume Romet-Lemonne, Michael Way, Carolyn A. Moores

**Affiliations:** 1 Institute of Structural and Molecular Biology, Birkbeck College, London, UK; 2 The Francis Crick Institute, London, UK; 3 Institut Jacques Monod, Université Paris Cité, CNRS, Paris, France; 4 Department of Infectious Disease, Imperial College, London, UK; 5 Present Address: Institut Jacques Monod, Université Paris Cité, CNRS, Paris, France

**Keywords:** Cryoelectron microscopy, Motility

## Abstract

Branched actin networks nucleated by the Arp2/3 complex have critical roles in various cellular processes, from cell migration to intracellular transport. However, when activated by WISH/DIP/SPIN90-family proteins, Arp2/3 nucleates linear actin filaments. Here we found that human SPIN90 is a dimer that can nucleate bidirectional actin filaments. To understand the basis for this, we determined a 3-Å-resolution structure of human SPIN90–Arp2/3 complex nucleating actin filaments. Our structure shows that SPIN90 dimerizes through a three-helix bundle and interacts with two Arp2/3 complexes. Each SPIN90 molecule binds both Arp2/3 complexes to promote their activation. Our analysis demonstrates that single-filament nucleation by Arp2/3 is mechanistically more like branch formation than previously appreciated. The dimerization domain in SPIN90 orthologs is conserved in metazoans, suggesting that this mode of bidirectional nucleation is a common strategy to generate antiparallel actin filaments.

## Abstract (web summary)

Liu et al. show that SPIN90 dimerizes and binds two Arp2/3 complexes to nucleate two bidirectional actin filaments and the dimerization domain is conserved in multicellular animals, suggesting that the mechanism of bidirectional actin filament nucleation is conserved.

## Main

**Extended Data Fig. 1:**
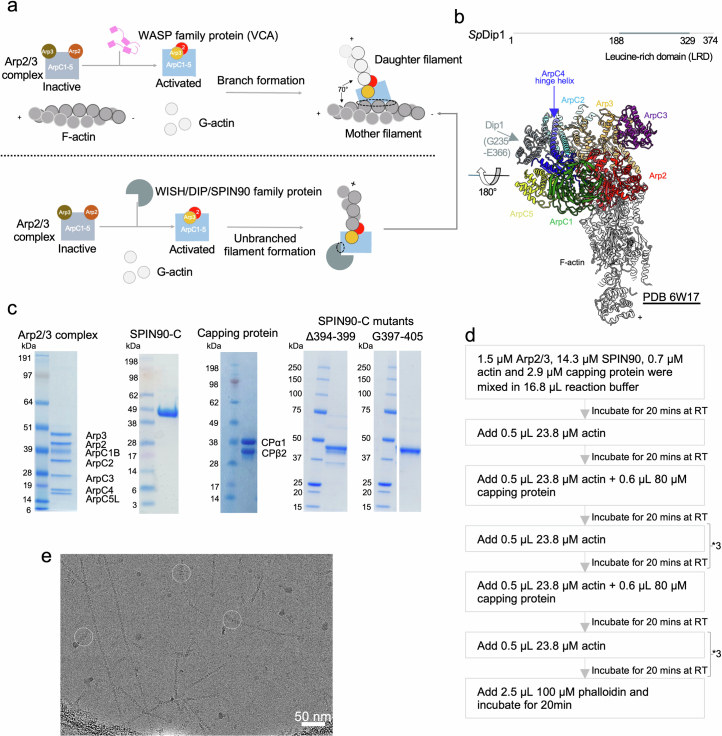
Overview of actin filament nucleation, purified proteins used in actin filament reconstitution, reconstitution workflow and exemplar cryo-EM micrograph. **a**. Schematic of Arp2/3-mediated actin branch formation and actin branch initiation in which the difference in interaction interface between mother filament and WISH/DIP/SPIN90 monomer are indicated with black dotted ovals. **b**. Top: *Sp*Dip1 domain organisation. Bottom: Complex structure of Dip1 and Arp2/3 complex at the end of their nucleated actin filament (PDB 6W17) shown in ribbon representation, with the resolved segment of Dip1 shown in grey-blue. **c**. SDS-PAGE gels showing purified proteins used in cryo-EM, pyrene assay and TIRF experiments, including purified proteins that had been used in a previous study of ours^26^. Similar protein quantity and quality were obtained from at least 2 independent purification batches. **d**. Flow chart showing how the cryo-EM sample was prepared. **e**. A representative cryo-EM image of SPIN90-Arp2/3-nucleated actin filaments. Particles used for data processing are highlighted in white circles. 10,235 micrographs with similar image quality were collected and processed. Scale bar = 50 nm. Source data

The actin cytoskeleton is a versatile dynamic assembly of filaments formed by the polymerization of actin monomers that participates in many cellular processes. A major feature of the actin cytoskeleton is branched actin networks, which generate the forces necessary for membrane deformation, intracellular trafficking and cell migration^1–5^. The Arp2/3 complex, consisting of seven subunits (Arp2, Arp3 and ArpC1–ArpC5), is the only known cellular constituent that can nucleate daughter actin filaments from the side of a pre-existing mother filament^1,6–12^. In the presence of pre-existing mother filaments, activation of Arp2/3 by class 1 nucleation-promoting factors (NPFs) induces a set of conformational changes within the complex that enable actin nucleation and thereby allow branch assembly^13–26^. These include twisting of the hinge helices in ArpC2 and ArpC4 that moves the actin-like Arp2 subunit toward Arp3 to produce an F-actin-like, short-pitch arrangement, with Arp2 and Arp3 each adopting a flattened structure^20,22,23,25,26^. Together, these conformational changes create a template for the addition of actin monomers and the nucleation of a daughter filament (Extended Data Fig. 1a). While recent advances have clarified multiple aspects of the mechanism of branch formation, a central question remains: how is the initial mother filament formed? Because actin monomers are in complex with profilin in cells, spontaneous nucleation of actin filaments is rare^27^. This points to the crucial role of actin filament nucleators in defining the timing and orientation of linear mother filament formation before initiation of the branched actin network.

**Fig. 1:**
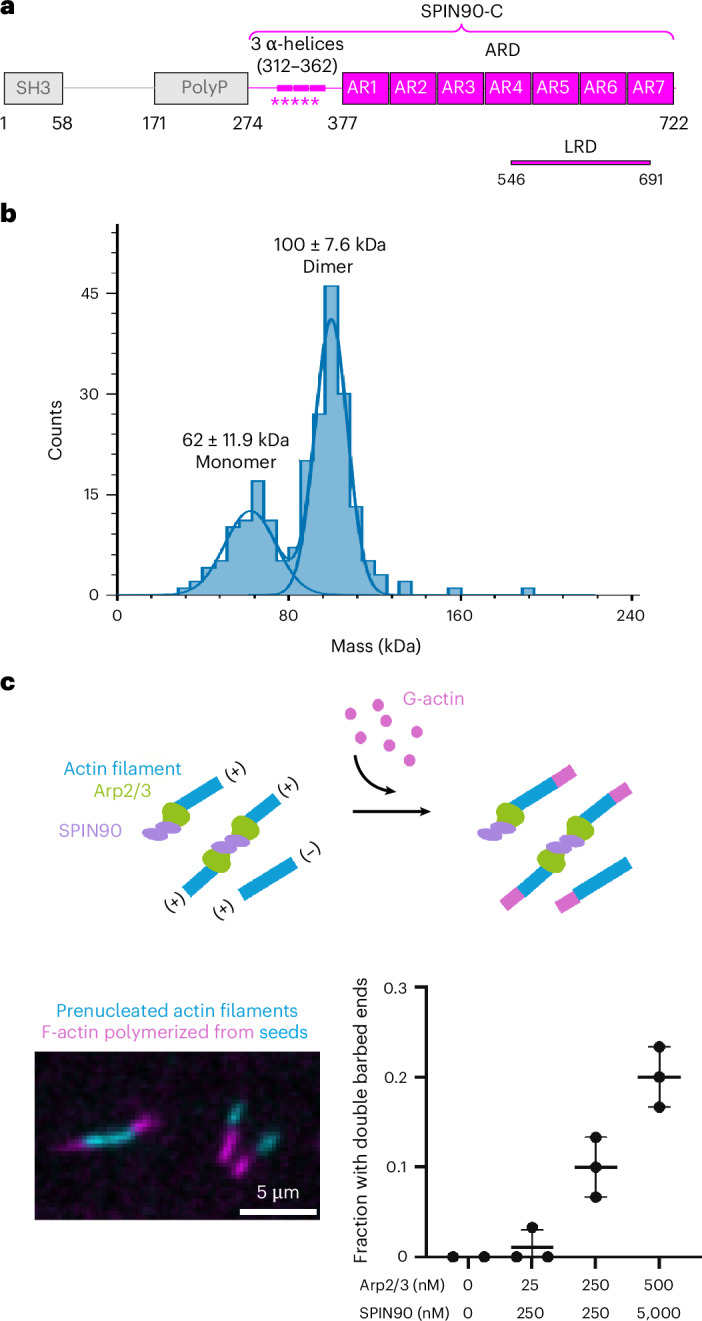
SPIN90 forms a dimer that induces bidirectional Arp2/3-nucleated actin filament polymerization. **a**, Human SPIN90 (Q9NZQ3) domain organization. The SPIN90-C construct used in this study is colored in magenta. The N-terminal region of SPIN90-C that is essential for activating the Arp2/3 complex in addition to the armadillo repeat domain (ARD) is indicated with magenta stars^32^. **b**, Mass distribution of 30 nM SPIN90-C, whose theoretical molecular weight is 50.0 kDa. Two peaks were observed with molecular weight corresponding to 100 ± 7.6 kDa and 62 ± 11.9 kDa, showing that SPIN90-C exists mainly as a dimer in solution. **c**, Top: schematic of in vitro TIRF microscopy assay to investigate the assembly of actin filaments from two ends of SPIN90-activated Arp2/3 complex. Prepolymerized actin filaments (15% labeled with Alexa-568, cyan) were mixed with G-actin (15% labeled with Alexa-488, magenta). Bottom left: the barbed ends were visualized directly by the Alexa-488 signal. Bottom right: quantification of fraction of actin filaments with double barbed ends. Each point represents the result of an independent experiment. The bars show the mean and s.d. of 2–3 independent replicates, each consisting of 30 filaments. Source data

In yeast, the actin regulator Dip1 can activate Arp2/3 without the need for preformed mother actin filaments^28^. Arp2/3-mediated actin patch formation and endocytosis in yeast are delayed in the absence of Dip1, highlighting its importance in generating initial mother filaments for branch network formation^29,30^. Dip1 is a member of the WISH/DIP/SPIN90 family, characterized by a conserved leucine-rich domain (LRD) that binds Arp2/3 (ref. ^28^; Extended Data Fig. 1b). In mammals, the SPIN90 LRD sits within a C-terminal armadillo repeat domain, while N-terminal SH3 and polyproline regions are involved in SPIN90 localization and interactions with the signaling adaptor Nck, respectively^31^ (Fig. 1a). Importantly, however, residues in the region that connect the polyproline region and the armadillo repeat domain (residues 274–376) are essential for activating the mammalian Arp2/3 complex^28,31,32^. The knockdown of mammalian SPIN90 leads to a loss of growth-factor-induced lamellipodia formation and EGFR-mediated endocytosis^33,34^. Moreover, SPIN90 depletion reduces cortical actin mesh size in blebs and stiffens the mitotic cortex, further highlighting its importance in regulating actin organization and dynamics^35^. Together, these findings suggest that SPIN90 also seeds branched actin networks in mammalian cells by generating mother filaments.

The cryo-electron microscopy (cryo-EM) structure of Dip1-activated yeast Arp2/3 at the end of a nucleated actin filament reveals that the LRD of Dip1 binds to the ArpC4 hinge helix^36^ (Extended Data Fig. 1b). The same interaction is also observed in the cocrystal structure of the human SPIN90 armadillo repeat domain bound to inactive bovine Arp2/3 (ref. ^32^). It was, therefore, proposed that WISH/DIP/SPIN90-family proteins activate Arp2/3 through a conserved mechanism that involves interacting with and bending the ArpC4 hinge helix. This in turn promotes the Arp2/3 short-pitch conformation necessary to nucleate an actin filament.

Although current structural insights have deepened our understanding of how WISH/DIP/SPIN90-family proteins interact with and activate the Arp2/3 complex, several questions remain. In particular, how does the small interface between WISH/DIP/SPIN90 proteins and ArpC4 fully activate Arp2/3, especially when activation during actin branch formation requires extensive interactions with the mother filament (Extended Data Fig. 1a)? Furthermore, what contribution do residues 274–376 N-terminal to the armadillo repeat domain of mammalian SPIN90 make to Arp2/3 activation? To address these questions, we examined the interaction of human SPIN90 with human Arp2/3 complex using biophysical and structural approaches. We found that SPIN90 forms a dimer through a three-helix domain N-terminal to the armadillo repeats, facilitating bidirectional actin filament nucleation by activating two Arp2/3 complexes. The three-helix domain and predicted dimeric structure are present in multicellular animals, suggesting that this mechanism of bidirectional actin filament nucleation is conserved in metazoans.

## Results

### SPIN90 dimers induce bidirectional Arp2/3-nucleated actin filament polymerization

To better understand how SPIN90 promotes Arp2/3-mediated actin nucleation, we first studied the biophysical properties of the minimal functional region of recombinant human SPIN90, which lacks its signaling and localization domains (SPIN90-C; Fig. 1a). Using mass photometry and 30 nM SPIN90-C, we observed a major peak with a molecular mass of 100 kDa, approximately corresponding to the size of a SPIN90-C dimer, and a smaller monomeric peak (Fig. 1b). To explore the implications of this unexpected SPIN90 dimerization for Arp2/3 activation, we performed total internal reflection fluorescence (TIRF) microscopy (Fig. 1c). We first polymerized short, fluorescently labeled actin seeds (labeled with Alexa-568, cyan) in the presence of SPIN90 and the Arp2/3 complex, then added actin monomers (labeled with Alexa-488, magenta) and visualized dynamic filament growth. Whereas activated Arp2/3 usually nucleates unidirectional actin polymerization, we found that, with increasing concentrations of SPIN90 together with Arp2/3, more actin polymerized from both ends of the actin seeds (Fig. 1c). This demonstrates that SPIN90 dimerization can drive bidirectional Arp2/3-mediated actin filament growth.

**Fig. 2:**
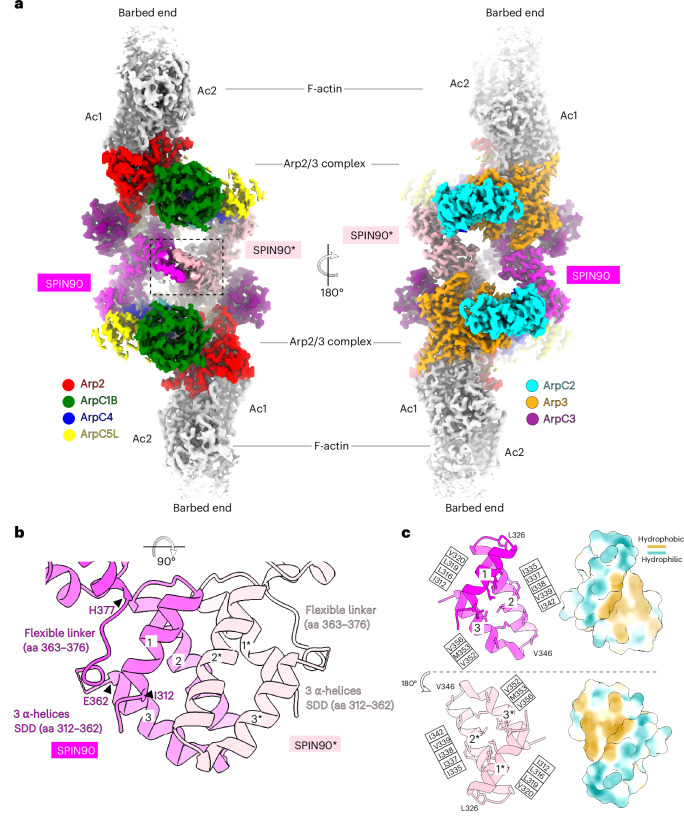
Structure of the SPIN90–Arp2/3-nucleated bidirectional actin filaments. **a**, Overview of the cryo-EM reconstruction of the SPIN90–Arp2/3 complex nucleated bidirectional actin filaments. Densities of individual proteins are colored according to the labels, and the nucleated actin filament subunits are colored gray and are labeled Ac1 and Ac2. **b**, Zoomed-in view of the three α-helices (residue 312–362) from the two SPIN90s mediating dimerization, referred to as the SDD; SPIN90 and SPIN90* are colored in magenta and pink, respectively. **c**, Open-book view (SPIN90* rotated 180° from the orientation in **b**) of the dimerization interface, showing that the SDD inserts into a hydrophobic groove on the SDD from the opposite SPIN90. Hydrophobic residues on the interaction surface of each SPIN90 are labeled and shown in stick representation. Right: hydrophobic surfaces are colored in yellow and hydrophilic regions are colored in teal.

**Extended Data Fig. 2:**
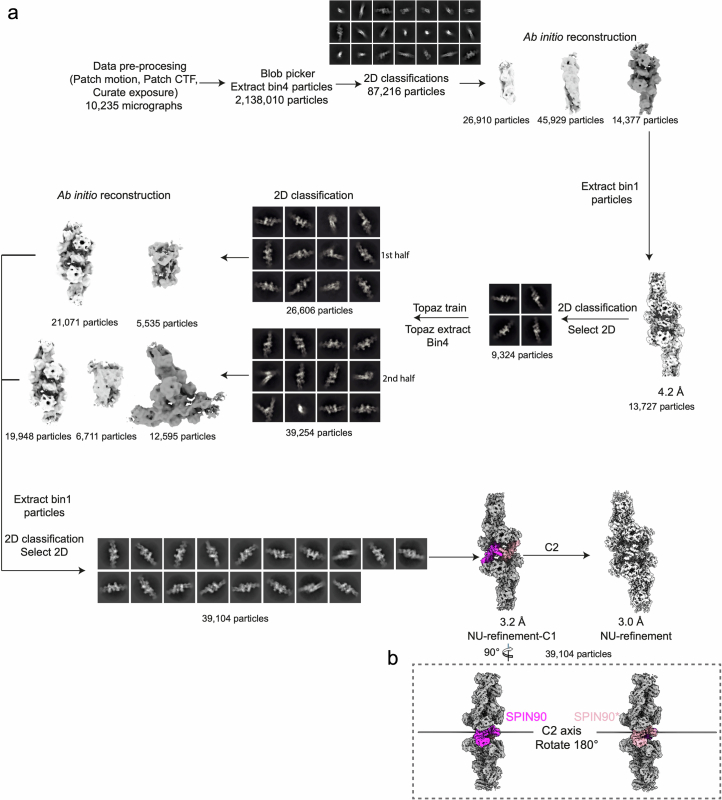
Image processing workflow for cryo-EM reconstruction. **a**. Data were processed using CryoSPARC. After initial pre-processing steps (Patch motion, Patch CTF and Curate exposure), 10,235 micrographs were selected for further processing. Three rounds of 2D classification were used to clean the dataset, followed by *ab initio* reconstruction with three classes. Particles from the class showing the characteristic ArpC1B β-propeller density were selected for 3D refinement. Subsequently, another round of 2D classification was used to further clean the data. The remaining 9,324 particles were used as a positive training set for Topaz particle selection. The Topaz-selected 65,860 particles were subjected to 2D classifications and *ab initio* reconstructions in two batches. A total of 39,104 particles were selected for the final reconstruction with C2 symmetry imposed. **b**. Two views of the C1 reconstruction with SPIN90 (magenta) and SPIN90* (pink) coloured. The C2 axis is indicated. Such an arrangement would facilitate one-sided interactions with signalling cascades at the plasma membrane via the SH3 domain and Poly-P domain, potentially aiding the activation process.

**Extended Data Fig. 3:**
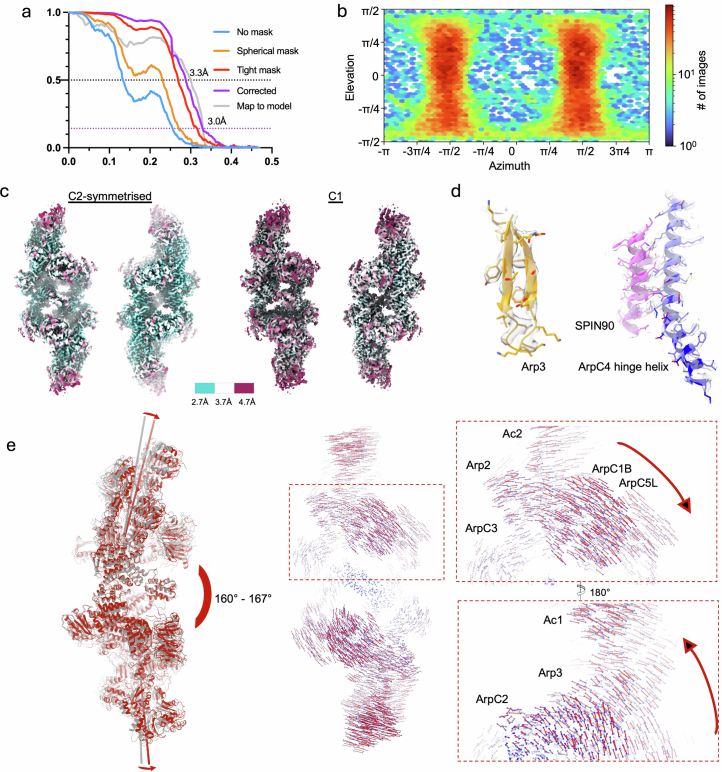
Cryo-EM validation and flexibility analysis of the cryo-EM reconstruction. **a**. Half-map and map to model Fourier Shell Correlation (FSC). Global resolution (FSC cut off 0.143) and map-model resolution (FSC cut off 0.5) are indicated. **b**. The angular distribution of particles used for the final 3D refinement. **c**. Cryo-EM symmetrised and C1 reconstruction coloured by estimated local resolution. **d**. Representative density with the fitted model showing the assignment of amino acid side chains. **e**. Left, views of two extreme models (shown in light grey and red) superimposed on the SPIN90 dimerisation domain (SDD). The helical axes of the bidirectional actin filaments in each model are displayed in the same colour as their corresponding models. The inter-filament angular range between the two bidirectional actin filaments is between 160° and 167°. Middle, displacement vectors (red) for Cα atoms (blue) between two extreme models obtained from the top variability mode analysis. Right, zoomed-in view of the boxed region in the left panel. Subunits and displacement directions are labelled. Source data

**Extended Data Fig. 4:**
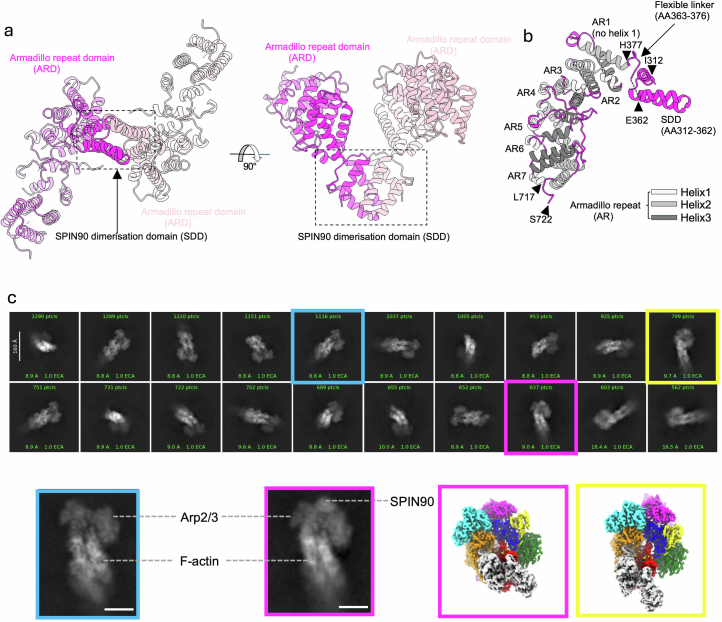
SPIN90 dimer and monomer organisation. **a**. Left, view of SPIN90 dimer shown in the same orientation and colour scheme as in Fig. 2a. Right, rotated view highlighting the three α-helices that mediate SPIN90 dimerisation. A zoomed-in view of the boxed region is shown in Fig. 2b. **b**. Detailed structural annotation of SPIN90. Armadillo repeats 1-7 are numbered. The α-helices within each repeat are coloured according to their position. **c**. 2D class averages showing density at the end of actin filament. The representative 2D class average in the blue box is in the similar orientation to that in the magenta box but reveals no SPIN90 density. The zoned maps from the dimeric reconstruction in this study (Fig. 2) in the magenta and yellow box, containing SPIN90 dimer, Arp2/3 complex and their nucleated unidirectional filament, are placed in orientations approximately corresponding to the boxed 2D class averages in the same colour. The zoned reconstructions illustrate that the view of SPIN90* in the corresponding 2D class averages means its oligomeric state is not visible. Scale bar = 50 Å.

To understand the mechanism of SPIN90 dimerization and its contribution to actin nucleation, we used cryo-EM to determine the structure of the SPIN90–Arp2/3 complex in the presence of actin (Table 1 and Extended Data Fig. 1c–e). The resulting reconstruction (at approximately 3-Å overall resolution (Extended Data Figs. 2 and 3a–d)) revealed a SPIN90 *C*_2_-symmetric dimer at its center, flanked by two activated Arp2/3 complexes nucleating actin filaments in a bidirectional fashion (Fig. 2a and Supplementary Video 1). Three-dimensional (3D) variability analysis of the *C*_1_ structure reveals that the bidirectional filaments are not perfectly antiparallel but rather exhibit an interfilament angular range of between 160° and 167° (Extended Data Fig. 3e). The variability analysis also revealed some stretching because of intrinsic flexibility of the SPIN90 armadillo repeats (Extended Data Fig. 3e and Supplementary Video 2), a well-known property of these extended, curved domains^37^. Our structure shows that the SPIN90 dimerization domain (SDD) is formed from three α-helices (residues 312–362) that interact through extensive rigid, hydrophobic contacts, with a buried surface area of 978 Å^2^ (Fig. 2b,c, Extended Data Fig. 4a and Supplementary Video 1). The SDD is connected by a flexible loop (residue 363–376) to the seven armadillo repeats (residues 377–717) (Figs. 1a and 2b and Extended Data Fig. 4a,b), while the N-terminal 38 residues of SPIN90-C are not visible, likely because of flexibility. Because unidirectional filaments are conspicuous in the TIRF experiment, we interrogated our cryo-EM data to extract unidirectional filaments (Methods). Two-dimensional (2D) averages of the resulting filament ends revealed that these particles have a strong preferred orientation such that there were insufficient views to determine a three-dimensional reconstruction. Nevertheless, they were detailed enough to show that the majority of these unidirectional filaments are capped at their point ends by Arp2/3, with no SPIN90 density visible (Extended Data Fig. 4c). Only around 20% of particles classified in 2D averages with evidence of SPIN90 binding but, even then, the projection view was such that it was not possible to assess whether the protein is a monomer or dimer. We think the most likely explanation for the features of these unidirectional filaments is that, under cryo-EM sample conditions, SPIN90 dimers can dissociate from activated Arp2/3, leaving single filaments capped by Arp2/3 at their pointed ends. As these data showed that the interaction between SPIN90 dimers and Arp2/3 is most readily visualized in the context of the bidirectional filaments, we focused our subsequent analysis on this complex.

**Table 1:** Cryo-EM data collection, refinement and validation statistics

	SPIN90–Arp2/3 complex in the presence of actin, (EMD-52580), (PDB 9I2B)
**Data collection and processing**	
Magnification	×81,000
Voltage (kV)	300
Electron exposure (e^−^ per Å^2^)	39.2
Defocus range (μm)	−0.9 to −2.4
Pixel size (Å)	1.06
Symmetry imposed	*C*_2_
Initial particle images (number)	2,138,010
Final particle images (number)	39,104
Map resolution (Å)	3.0
FSC threshold	0.143
Map resolution range (Å)	2.7–4.7
**Refinement**	
Initial model used (PDB code)	8P94, 6DEE
Model resolution (Å)	3.3
FSC threshold	0.5
Map sharpening *B* factor (Å^2^)	−62.8
Model composition	
Non-hydrogen atoms	90,796
Protein residues	6,128
Ligands	8 Mg^2+^, 8 ADP, 4 phalloidin
*B* factors (Å^2^)	
Protein	36.44
Ligand	37.2
Root-mean-square deviations	
Bond lengths (Å)	0.007
Bond angles (°)	0.839
Validation	
MolProbity score	1.21
Clashscore	2.52
Poor rotamers (%)	0
Ramachandran plot	
Favored (%)	97
Allowed (%)	3
Disallowed (%)	0

FSC, Fourier shell correlation.

**Extended Data Fig. 5:**
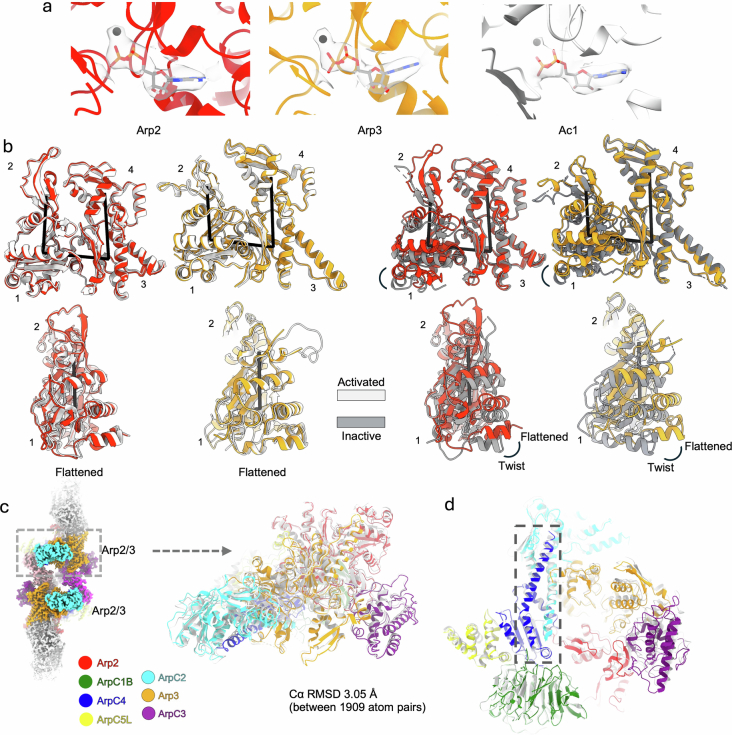
Nucleotide occupancy and demonstration of active state of the Arp2/3 complex. **a**. Density (transparent) from the symmetrised reconstruction and models of ADP (in stick representation) and Mg2+ (black dot) in Arp2, Arp3 and actin subunit Ac1. **b**. Left, Superimposition of SPIN90-activated Arp2 and Arp3 with branch junction-activated Arp2 and Arp3 (PDB 8P94). Right, Superimposition of SPIN90-activated Arp2 and Arp3 with inactive Arp2 and Arp3 (PDB 6DEC). Each superimposition is shown in two orientations. Structures are overlayed on subdomain 3 and 4. The twist angle is the dihedral angle between the centres of mass of each of the four subdomains, plotted in Fig. 5a. **c**. Structural comparison of the SPIN90-activated Arp2/3 complex (this manuscript, subunits coloured) and the Arp2/3 complex at the branch junction (PDB 8P94, subunits in light grey). Structures were aligned on Arp3 subdomain 1 and 2 (AA6-37, AA60-153 and AA375-409). Overall Cα RMSD is shown. **d**. Rotated sliced view of the structural comparison but aligned on ArpC4 globular domain (AA2-141). A zoomed-in view of the boxed region is provided in Fig. 3b, showing the similar ArpC4 hinge helix twist angle between the two structures.

**Extended Data Fig. 6:**
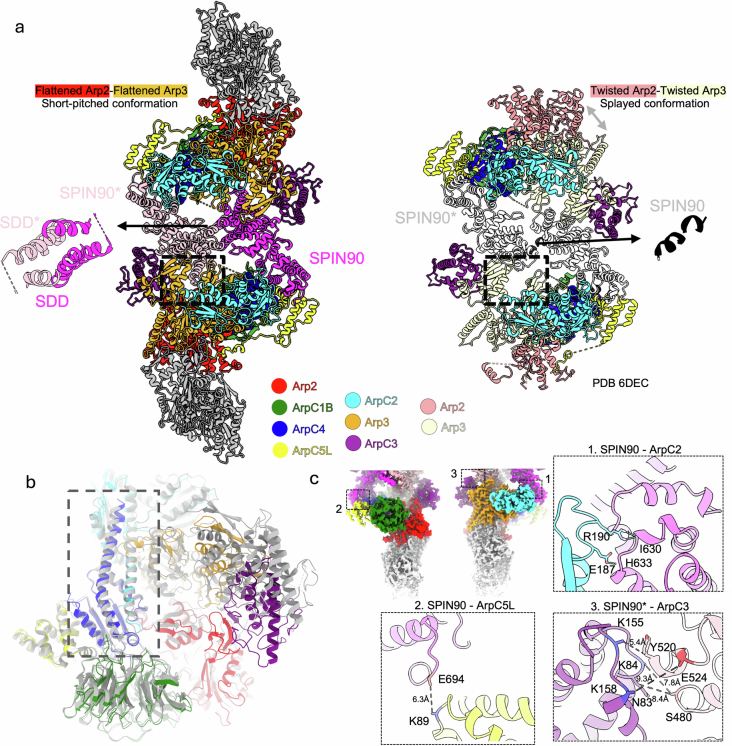
Structural comparison of the SPIN90-activated Arp2/3 complex with its nucleated actin filaments and SPIN90-inactive Arp2/3 co-crystal structure. **a**. Left, model of the SPIN90-activated Arp2/3 complex with its nucleated actin filaments (left). Arp2/3 subunits and SPIN90 dimer are coloured-coded as in Fig. 2a with the nucleated actin filaments shown in grey. A zoomed-in view highlights the three α-helices (SDD) from both SPIN90 molecules. Arp2 and Arp3 are flattened (activated, detailed in Extended Data Fig. 5b) and arranged in a short-pitched conformation. The structural details in the dashed black box are analysed in Fig. 3. Right, SPIN90-inactive Arp2/3 co-crystal structure (PDB 6DEC). Arp2/3 subunits are coloured as in Fig. 2a, except for Arp3, which is coloured in dark salmon, and Arp2, coloured in pale goldenrod. SPIN90 is coloured in light grey. There are two short α-helices at the location of the three α-helices in the left panel, coloured in black. Arp2 and Arp3 are twisted (inactive, detailed in Extended Data Fig. 5b) and adopt a splayed conformation. The structural details in the black box are analysed further in Extended Data Fig. 7a. **b**. Structural comparison of the SPIN90-activated Arp2/3 complex (this manuscript, subunits coloured) and the Arp2/3 complex at the above SPIN90-inactive Arp2/3 co-crystal structure (PDB 6DEC, subunits in light grey). Structures were aligned on ArpC4 globular domain (AA2-141). Zoomed-in view of the boxed region is shown in Fig. 3b, showing the ArpC4 hinge helix twist angle difference between the two structures. **c**. Zoomed-in view of the minor interfaces between the SPIN90 dimer and Arp2/3 complex subunits. Residues involving hydrogen bonds (blue dotted line) and the potential salt bridges (black lines) are shown in stick representation. Hydrogen bonds were detected by PISA. Potential electrostatic interactions were inferred based on Cβ–Cβ distance within 10 Å between polar residues where the side chain is not resolved.

In our bidirectional filament reconstruction, Arp2 and Arp3, which are bound to adenosine diphosphate (ADP) (Extended Data Fig. 5a), are both flattened and adopt a short-pitch conformation (Extended Data Fig. 5b). Comparison of the Arp2/3 complexes in our reconstruction to those at branch junctions, confirmed that they are in an active conformation (Extended Data Fig. 5c,d). The previous X-ray crystallography structure of SPIN90 (residues 269–722) was determined in complex with Arp2/3 in an inactive conformation^32^. The residues that form the SDD were present in the crystallized construct but were insufficiently ordered to unambiguously determine their structure; thus, dimerization was not visualized (Extended Data Fig. 6a). Intriguingly, however, the overall organization of the SPIN90–Arp2/3 complex within the crystallographic unit cell is similar to our dimeric structure with active Arp2/3, suggesting that SPIN90 did indeed form dimers but the crystal context inhibited complex activation. This validates the idea that SPIN90 readily dimerizes but also suggests that the SPIN90 dimer structure is most stable and, therefore, most readily visualized in complex with activated Arp2/3.

### The interaction between SPIN90 and Arp3 is essential for Arp2/3 activation

**Fig. 3:**
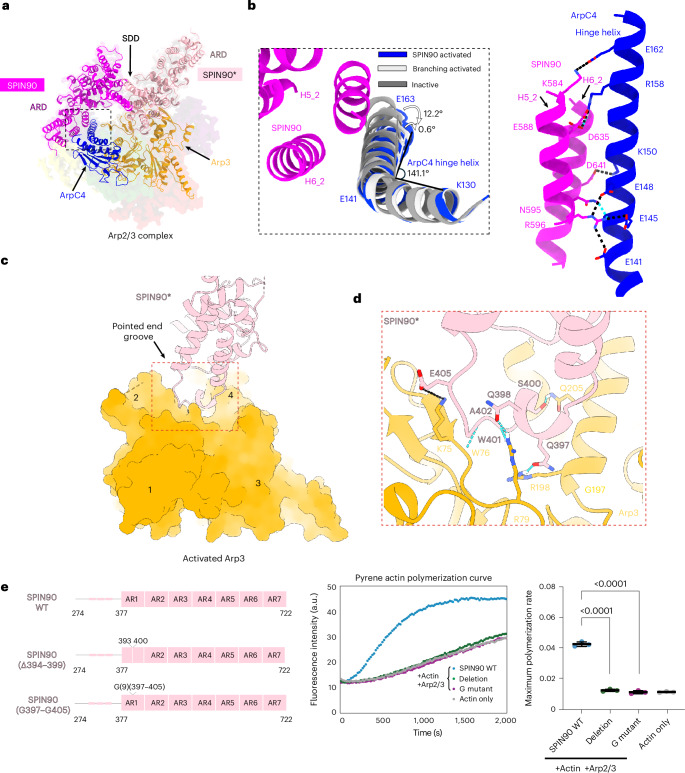
Dimeric SPIN90 forms two interfaces with each activated Arp2/3 complex to promote actin nucleation. **a**, Overview of two major interactions between the SPIN90 dimer and a single Arp2/3 complex. The SPIN90 dimer, ArpC4 and Arp3 are modeled and shown in ribbon representation within the cryo-EM map. The cryo-EM map is displayed with transparency to highlight the interactions. The color scheme is the same as in Fig. 2a. **b**, Left: comparison of the SPIN90-bound ArpC4 hinge helix to the branch structure (PDB 8P94) and the inactive state (PDB 6DEC). Structures are aligned on the basis of ArpC4 residues 2–141. The ArpC4 bending angle is defined using K130 Cα, E141 Cα and E163 Cα. Right: interactions between SPIN90 (H5_2, the second helix within the fifth armadillo repeat; H6_2, the second helix within the sixth armadillo repeat) and the ArpC4 hinge helix. Right: residues forming hydrogen bonds (blue dotted line) and the salt bridge (black line) are shown in stick representation. **c**, Overview of the interaction between SPIN90* and Arp3. SPIN90 is shown in ribbon representation while Arp3 is displayed in surface representation. The subdomains in Arp3 are labeled. **d**, Zoomed-in view of the interface between SPIN90* and Arp3 pointed-end groove inside the red box in **c**. Residues forming hydrogen bonds (blue dotted line) and the salt bridge (black line) are shown in stick representation. **e**, Left: schematic of SPIN90 Arp3 interaction mutants. Representative assay curves (middle) and quantification (right) of the maximum polymerization rate for pyrene assay reactions in the presence of Arp2/3 complex and actin. The data for actin alone correspond to the maximum spontaneous polymerization rate. Εach point represents the maximum polymerization rate of a pyrene curve. The bar indicates the mean of three repeats and the error bar shows the s.d. A two-sided unpaired *t*-test was applied to analyze the statistical significance, with *P* values shown on the top (WT versus deletion mutant, *P* < 0.0001; WT versus G-mutant, *P* < 0.0001). Source data

To investigate how SPIN90 dimerization promotes Arp2/3 complex activation, we analyzed the interactions between the SPIN90 dimer (with each monomer referred to as SPIN90 and SPIN90*) and one of the Arp2/3 complexes. SPIN90 and SPIN90* contact each Arp2/3 complex through two distinct interfaces (Fig. 3a and Supplementary Video 3). The first, as previously defined, involves a 936-Å^2^, primarily electrostatic interaction of the armadillo repeat domain of SPIN90 with the ArpC4 hinge helix (Fig. 3a,b and Supplementary Video 3). Adjacent to this, smaller (322 Å^2^) hydrogen-bond contacts are also made with ArpC2, while 150 Å^2^ of buried surface area with ArpC5L was calculated, likely forming an electrostatic interaction (Extended Data Fig. 6c). Our structure shows that the ArpC4 hinge helix conformation matches that in the branch junction structure (Extended Data Fig. 5d) but is bent compared to that in the SPIN90-inactive Arp2/3 cocrystal structure (Fig. 3b and Extended Data Fig. 6b). This shows that bending of the ArpC4 helix is a common feature of Arp2/3 complex activation, both at branches through mother filament interaction and in linear filament nucleation by SPIN90 (Fig. 3b).

**Extended Data Fig. 7:**
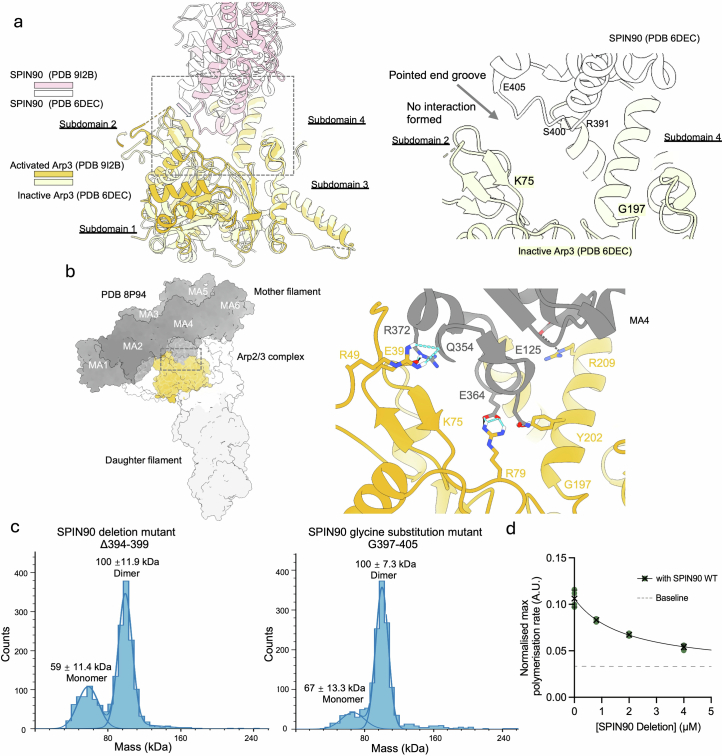
The interaction between the SPIN90 and Arp3 is unique to the activated conformation of Arp3. **a**. Left: Overlay of the models containing SPIN90 and Arp3 (Extended Data Fig. 5). The models are aligned on the subdomains 3 and 4 of the Arp3. Subdomains in Arp3 are labelled. Right: In the SPIN90-inactive Arp2/3 co-crystal structure (grey box on the left and black box in Extended Data Fig. 5, right panel), approximately half of the SPIN90 loop is unresolved due to its flexible nature, while the other half is positioned away from the Arp3 pointed-end groove, resulting in a loss of stable interactions. The Arp3 pointed end groove is indicated with a black arrow. End residues and breaking points of the SPIN90 loop are labelled. **b**. Left: Actin branch junction structure is shown in surface representation (PDB 8P94). The mother filament is coloured in dark grey. The daughter filament is coloured light grey. Arp3 is shown in orange while other Arp2/3 subunits are shown in transparency. The mother filament subunits are labelled with MA1-MA6. Right: The cropped zoomed-in view of the interaction between MA4 and Arp3. Residues forming hydrogen bonds (blue dotted line) and the salt bridge (black line) are shown in stick representation. **c**. Mass distribution of 30 nM SPIN90 mutant molecule, whose molecular weight are around 50 kDa respectively in theory. For the deletion mutant, two peaks were observed with molecular weight corresponding to 100 ± 11.9 kDa and 59 ± 11.4 kDa. For the G loop mutant, two peaks were observed with molecular weight corresponding to 100 ± 7.3 kDa and 67 ± 13.3 kDa. **d**. Plot of maximum polymerisation rate versus concentration of SPIN90 mutant for pyrene actin polymerisation assays containing 0.4 μM WT SPIN90, 8 nM Arp2/3 complex, 2.5 μM 5% pyrene‐labelled actin and the indicated concentration of the SPIN90 mutant. The cross indicates the mean of three repeats and the error bar shows the standard deviation. Source data

The second, previously uncharacterized but equivalently sized interface (buried surface area = 1,081 Å^2^) forms between the N-terminal end of the armadillo repeats of SPIN90* and Arp3 (Fig. 3a and Supplementary Video 3), with an additional minor contact with ArpC3 (buried surface area = 362 Å^2^) (Extended Data Fig. 6c). In this second interface, the interacting loop from SPIN90* (residues 393–406) inserts into the pointed-end groove of activated Arp3, forming electrostatic and hydrogen bonds (Fig. 3c,d). Moreover, the interaction between SPIN90* and Arp3 is unique to the activated conformation of Arp3 (Extended Data Fig. 7a). This is reminiscent of the role of the mother filament in stabilizing the active conformation of Arp3 during branch formation (Extended Data Fig. 7b), which promotes binding by the first actin subunit of the nucleated filament in both cases^23^. Thus, we hypothesize that this second SPIN90 interaction stabilizes Arp3 in an activated state, enabling the Arp2/3 complex to nucleate an actin filament (Supplementary Video 4).

To test this hypothesis, we generated two SPIN90 mutants: a deletion (Δ394–399) that shortens the Arp3 interaction loop and a glycine substitution (G397–G405) that alters the structural properties of the interacting loop (Fig. 3e). Mass photometry confirmed that both mutants still form dimers (Extended Data Fig. 7c) but neither mutant could activate the Arp2/3 complex (Fig. 3e). Furthermore, we found that titration of the Δ394–399 mutant into a pyrene assay reduced wild-type (WT) SPIN90–Arp2/3-mediated actin polymerization (Extended Data Fig. 7d), demonstrating that the mutant competed with WT SPIN90 to bind Arp2/3. Together, these findings demonstrate that the interactions of the armadillo repeat domain of SPIN90 with ArpC4 and with active Arp3 are essential for Arp2/3 complex activation.

### SPIN90 dimers and branch junctions bind active Arp2/3 through a similar mechanism

**Fig. 4:**
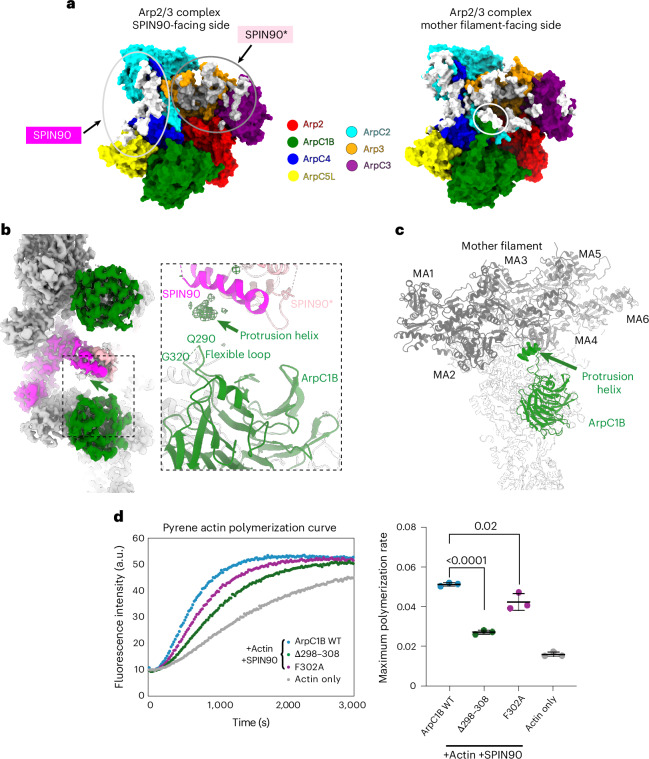
SPIN90 dimers and branch junctions bind active Arp2/3 through a similar mechanism. **a**, Interaction surface comparison. The interfaces are defined by residues that are within 5 Å of the SPIN90 dimer or the mother filament (PDB 8P94). The SPIN90 interface is colored in white and enclosed within the light-gray circle, and the SPIN90* interface is colored in gray and enclosed within the gray circle. The mother filament interface is colored in white, with the interface on ArpC1B protrusion helix highlighted in the white circle. Structures are rendered in space-filling representation to more effectively convey the contours of the binding surfaces. **b**, Left: overview of the cryo-EM reconstruction of the SPIN90–Arp2/3 complex nucleated bidirectional actin filaments. Right: cropped zoomed-in view of the model. The ArpC1B subunit is colored in green, SPIN90 is colored in magenta and pink, and other models are shown in light gray. In the zoomed-in view, the density attributed to the ArpC1B protrusion helix is represented as a difference in density. This difference density, shown in mesh representation, was calculated by subtracting the simulated 5-Å-resolution density of the model from the cryo-EM reconstruction using the ChimeraX ‘volume subtract’ command. **c**, Cropped zoomed-in view of the cortactin-bound actin branch junction (PDB 8P94). Only the cryo-EM density of the ArpC1B protrusion helix density is shown and indicated by the green arrow. The ArpC1B model is colored in green. The mother filament subunits are colored in dark gray and labeled with MA1–MA6. Other subunits are shown in light gray. **d**, The representative curves (left) and quantification of the maximum actin polymerization rate (right) for pyrene assay reactions containing the WT Arp2/3 complex, the Arp2/3 complex with the ArpC1B protrusion helix deleted, and the Arp2/3 complex containing the ArpC1B-F302A mutant in the presence of SPIN90. Each point represents the results of an independent experiment. The mean of the maximum polymerization rate and the s.d. of three independent replicates are shown. An unpaired two-sided *t*-test was used to calculate the *P* values, which are displayed in the figure (WT versus F302A, *P* = 0.02; WT versus ΔAA298–308, *P* < 0.0001). Source data

Comparison of the interface of activated Arp2/3 bound to the SPIN90 dimer to that of Arp2/3 on the mother filament at branch junctions reveals striking similarities in subunit contacts (Fig. 4a, white and gray surfaces). While the Arp2/3 buried surface area with the SPIN90 dimer is smaller than with the mother filament (2,791 Å^2^ versus 4,406 Å^2^), this is substantially larger than was indicated by previous structural analyses of the SPIN90–Arp2/3 interaction (~1,200 Å^2^)^32^ and demonstrates that the previous proposal that SPIN90 stabilizes activated Arp2/3 through a very small interaction with ArpC4 is incomplete. Rather, SPIN90 dimerization generates a twofold increase in the interaction area that stabilizes activated Arp2/3. Our observations demonstrate that both branch and single-filament nucleation are mechanistically more similar than previously appreciated in that they involve interactions with both ArpC4 and Arp3.

**Extended Data Fig. 8:**
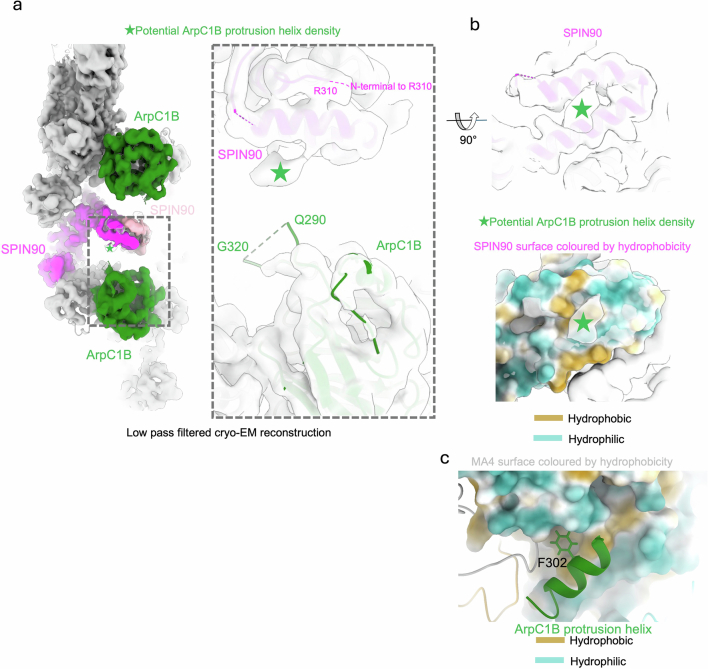
SPIN90 forms a loose interaction with the ArpC1B protrusion helix in the activated Arp2/3 complex. **a**. Complex overview (left) and zoomed-in view (right) of the undefined density beneath the SDD and adjacent to ArpC1B. Reconstruction was filtered using ‘volume gaussian’ in ChimeraX (with a standard deviation of 1 Å) to reduce noise and bring out the shape of the undefined density. **b**. Top, rotated view showing the undefined density highlighted with a green star. Bottom, the modelled SPIN90 surface is coloured based on hydrophobicity showing a hydrophobic patch facing the potential ArpC1B protrusion helix density, highlighted with a green star. Hydrophobic regions are coloured in yellow and hydrophilic surface regions are coloured in teal. **c**. Surface representation of the MA4 subunit in the mother filament at the actin branch junction, coloured by hydrophobicity. The interacting ArpC1B protrusion helix is shown in the ribbon representation. The hydrophobic F302 is also highlighted with stick representation.

One point of difference between the interaction surface of Arp2/3 with the mother filament at branches compared to the SPIN90 dimer is that the ArpC1B protrusion helix, which contacts the mother filament, is not apparent in the SPIN90 complex. On closer inspection, however, our structure revealed the presence of lower-resolution density contacting the SDD that we attribute to the ArpC1B protrusion helix (Fig. 4b and Extended Data Fig. 8a). The flexibility of the long loop in which the protrusion helix is embedded would allow its contact with SPIN90 (Fig. 4b). In addition, the position and properties of the predicted ArpC1B protrusion helix would enable an interaction with a hydrophobic groove on the surface of the SDD (Extended Data Fig. 8b).

In the branch junction structure, the ArpC1B protrusion helix inserts a conserved phenylalanine into the hydrophobic groove of the mother filament^23^ (Fig. 4c and Extended Data Fig. 8c). To investigate whether the contact of the ArpC1B protrusion helix with the SDD is required for SPIN90 to activate Arp2/3, we generated complexes lacking the helix or with an F302A substitution. We found that Arp2/3 complexes lacking the ArpC1B protrusion helix had significantly reduced actin nucleating activity (Fig. 4d). The F302A mutant also showed reduced activity albeit to a lesser extent, consistent with the idea that other residues in the protrusion helix contribute to its interaction with the SDD (Fig. 4d). Taken together, our observations suggest that the ArpC1B protrusion helix also functions in SPIN90-mediated activation of Arp2/3 to increase the interaction between SPIN90 and the Arp2/3 complex, albeit in a more flexible and dynamic way compared to the mother filament.

### Species-specific differences in Arp2/3 activated by the WISH/DIP/SPIN90 family

**Fig. 5:**
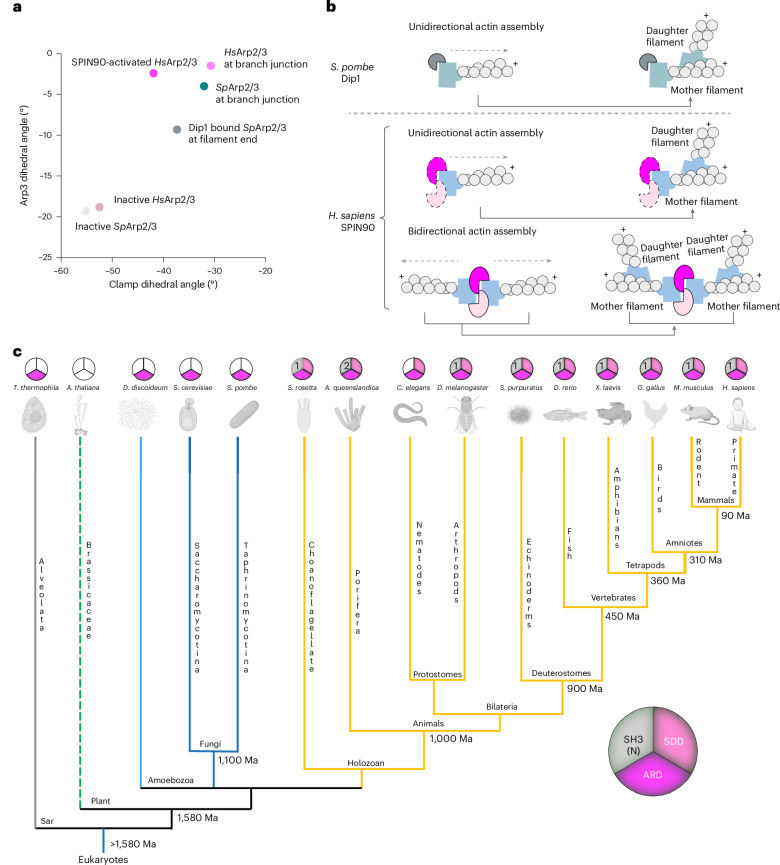
Conservation of dimerization in metazoan SPIN90 orthologs suggests a common mode of antiparallel actin filament formation. **a**, Plot of the clamp dihedral angle versus Arp3 dihedral angle in representative published Arp2/3 structures (Extended Data Fig. 9a). The clamp-twist angle was defined as the dihedral involving K18 Cα (ArpC2), I244 Cα (ArpC2), S147 Cα (ArpC4) and R32 Cα (ArpC4) in the human Arp2/3 complex or R18 Cα (ArpC2), I262 Cα (ArpC2), S147 Cα (ArpC4) and R32 Cα (ArpC4) in the metazoan Arp2/3 complex. The Arp3 dihedral angle is identified as the dihedral involving the centers of mass of subdomain 2, subdomain 1, subdomain 3 and subdomain 4. **b**, Schematic comparing the mechanisms of Dip1 monomer and SPIN90 dimer Arp2/3 activation to generate mother filaments for branch formation, with bidirectional mother filament organization enabling distributed daughter filament organization. Dip1 is shown in gray and blue, and metazoan Arp2/3 is shown in light gray and blue; the SPIN90 dimer is colored in magenta and pink, and human Arp2/3 is colored in light blue. The SPIN90 dimer at the end of the unidirectional actin filament was not directly observed in this study and is, therefore, indicated with dotted lines. **c**, Phylogenetic analysis of SPIN90 homologs in 15 model eukaryote organisms. The pie chart illustrates the domain organization of human SPIN90, divided into three sections representing the three major domains (SH3 domain, SDD and ARD). In SPIN90 homologs, a colored slice indicates the presence of the corresponding domain, whereas an uncolored slice indicates its absence, identified using AlphaFold predictions. The number in the SH3 domain section specifies the number of SH3 domains present. The phylogenetic tree indicates the model organisms’ relationships and estimated divergence time (Ma, million years ago)^46^. Please note that the branch lengths do not reflect evolutionary time. *A. queenslandica*, *Amphimedon queenslandica*; *A. thaliana*, *Arabidopsis thaliana*; *D. melanogaster*, *Drosophila melanogaster*; *D. rerio*, *Danio rerio*; *G. gallus*, *Gallus gallus*; *H. sapiens*, *Homo sapiens*; *M. musculus*, *Mus musculus*; *S. cerevisiae*, *Saccharomyces cerevisiae*; *S. pombe*, *Schizosaccharomyces pombe*; *S. purpuratus*, *Strongylocentrotus purpuratus*; *S. rosetta*, *Salpingoeca rosetta*; *X. laevis*, *Xenopus laevis*. Model organism schematics created with BioRender.com.

**Extended Data Fig. 9:**
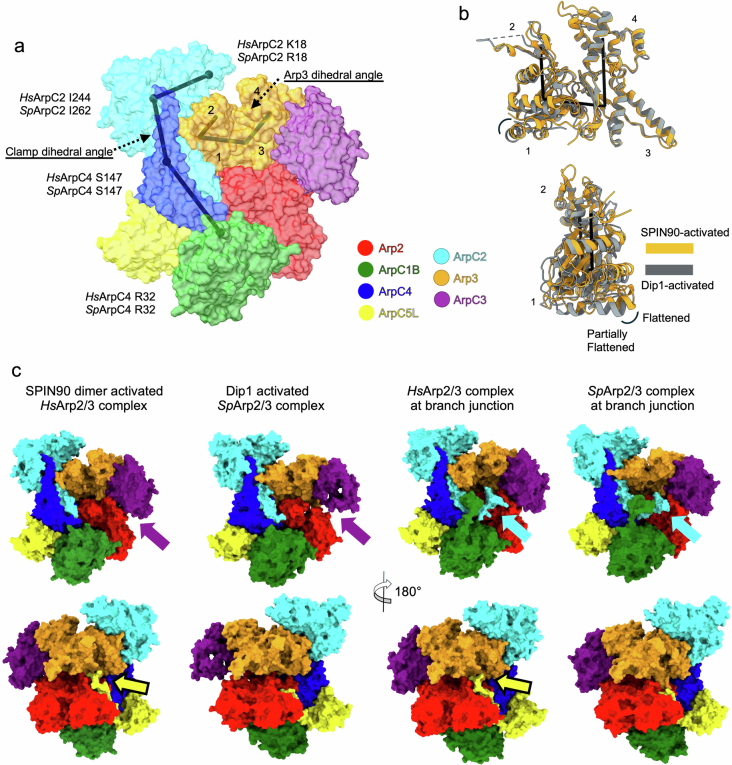
Comparison of the structures and interactions in activated human and yeast Arp2/3 complexes. **a**. Arp2/3 surface representation highlighting dihedral angle measurements for clamp twisting (short pitch formation) and Arp3 flattening in Fig. 5a and Extended Data Fig. 5. **b**. Superimposition of SPIN90-activated Arp3 with partially activated Arp3 by Dip1 (PDB 6W17) shown in two orientations. Structures are overlayed on subdomain 3 and 4. The twist angle is the dihedral angle between the centres of mass of each of the four subdomains, plotted in Fig. 5a. **c**. Comparison of differently activated human (Hs) and yeast (Sp) Arp2/3 complexes. Arp2/3 complexes are shown in surface representation to more effectively show subunit packing. Individual proteins are coloured according to the labels. The purple arrows highlight that the contacts between Arp2 and ArpC3 are absent in the Dip1 nucleated yeast Arp2/3 complex structure. The yellow arrows point to the insertion of the ArpC5(L) N-terminus into the groove formed by Arp2 and Arp3 only observed in human Arp2/3 complex structures. The N-terminus of *Sp*ArpC5 is 7 residues shorter and therefore cannot form these contacts. The blue arrows reveal the specific feature of ArpC2 only observed in branch junction structures, mediating the interaction of the Arp2/3 complex with the mother filament.

Having investigated the mechanism by which the SPIN90 dimer stabilizes activated mammalian Arp2/3, we compared our SPIN90-bound Arp2/3 structure to monomeric yeast Dip1–Arp2/3, with reference to Arp2/3 at actin branches. A striking difference between the SPIN90-activated human Arp2/3 and the Dip1-activated yeast complex relates to the flattening of Arp3 (refs. ^22,23,36^). In our SPIN90-activated human complex, both Arp2 and Arp3 are fully flattened with a dihedral angle of −2.5° for Arp3, which represents a fully active state primed for nucleation (Fig. 5a and Extended Data Fig. 9a). In contrast, the Dip1-activated yeast Arp2/3 exhibits partial flattening of Arp3, with a dihedral angle of −9.3° (ref. ^36^; Extended Data Fig. 9b). The complete flattening seen in our structure can be explained by the additional interaction between the SPIN90 dimer and Arp3. In addition, in the Dip1-actived Arp2/3 structure, ArpC3 does not contact Arp2 (Extended Data Fig. 9c, purple arrow), whereas, in the SPIN90-activated complex, ArpC3 connects Arp2 and Arp3, likely because of the full flattening of Arp3. Moreover, the extended N terminus of human ArpC5L inserts between Arp2 and Arp3 in the SPIN90-activated Arp2/3 structure in contrast to the shorter yeast ArpC5 N terminus that only contacts Arp2; these differences are also observed in the actin branch junction structures^22^^,26^ (Extended Data Fig. 9c, yellow arrow). Overall, our analyses indicate general differences in the properties of Arp2/3 complexes from human and yeast that are reflected in the mechanisms by which they are activated.

## Discussion

Using in vitro reconstitution and biophysical analyses, we discovered that human SPIN90 forms a dimer that activates two Arp2/3 complexes to nucleate bidirectional actin filaments (Fig. 5b). Moreover, our analysis demonstrates that the mechanisms by which mammalian Arp2/3 binds mother filaments at branches and at the end of linear filaments are more similar than previously thought. The SPIN90 dimer forms two interfaces with each Arp2/3 complex, binding to both the bent ArpC4 hinge helix and the pointed end of activated Arp3. We infer that dimerization, by increasing the total buried surface in the complex, contributes to stabilization of the activated state of human Arp2/3 complex. In contrast, in the yeast Arp2/3 complex, which has a lower energy barrier for activation, we suggest that both interactions are not required^18^^,19^^,38^ (Fig. 5b). The higher energy barrier for human Arp2/3 activation also explains why SPIN90 has evolved to form a dimer to enhance its activation ability compared to its yeast counterpart Dip1. These observations emphasize the structural plasticity of the Arp2/3 complex between human and yeast and show how WISH/DIP/SPIN90-family proteins and presumably other Arp2/3 regulators have evolved to accommodate the conformational variability of their cognate Arp2/3 complexes.

If the bidirectional actin filaments we observed were to act as mother filaments for subsequent actin branch initiation and propagation, then their bidirectionality would have important implications for the architecture of the resulting actin network. First, the organization of the SPIN90 dimer is predicted to position the SH3 and polyproline domains of each protein on the same side of the nucleating complex (Extended Data Fig. 2b). Such an arrangement would facilitate one-sided interactions with signaling cascades at the plasma membrane, potentially aiding the activation process. Second, the multivalent interactions involving Nck, NPFs and SPIN90 would give the branch formation process a robust jump start^30^. Once the mother filaments are nucleated, the class 1 NPFs can immediately promote the daughter filament assembly. Interestingly, Nck could interact with both SPIN90 and N-WASP through its three SH3 domains and thereby couple linear and branched actin filament nucleation^39,40^. Lastly, bidirectional organization, albeit with an interfilament angle of 160°–167°, can enable efficient actin branch formation as daughter filaments can be nucleated in opposite orientations, distributing the force for cellular processes such as cell migration and phagocytosis^41^ (Fig. 5b). The overall flexibility of SPIN90–Arp2/3 complex (Supplementary Video 2) may also contribute to the stability of SPIN90–Arp2/3-nucleated filaments in the presence of piconewton forces^42^. In addition, phosphorylation by Src family kinases is known to modulate cellular localization and function of SPIN90, suggesting that post-translational regulation of SPIN90 dimerization and unbinding of SPIN90 from activated Arp2/3 are likely to be points of additional control for the cellular actin network^43^. Future in vitro and cell-based studies together with mathematical modeling will be required to determine precisely how SPIN90-mediated bidirectional filament nucleation might enhance the efficiency of branched actin propagation, membrane modulation and response to external forces.

**Extended Data Fig. 10:**
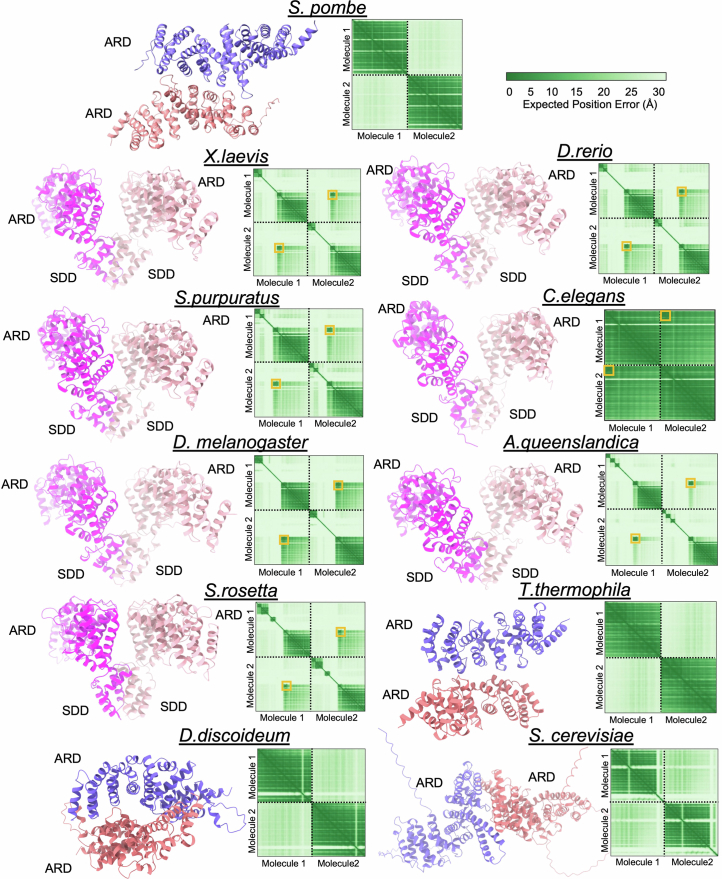
Structural prediction of dimerisation capabilities of WISH/DIP1/SPIN90 family proteins from representative model organisms. For each protein, the left panel shows the predicted AlphaFold model for the SDD and armadillo repeat domains. Subunits in each predicted dimer are coloured differently. The right panel for each protein shows a plot of predicted aligned error (PAE). The SDD domain regions of metazoan with low PAE are highlighted in orange boxes to indicate the confidence of dimer prediction. Note the high PAE between subunits in *S.pombe*, *S. cerevisiae*, *T. thermophila* and *D. discoideum* indicating the low probability of forming a stable dimer.

The differences in the structures of Dip1 and SPIN90-activated Arp2/3 complexes between human and yeast and their activation mechanisms prompted us to investigate conservation of the SPIN90 family more broadly. Comparison of SPIN90 homologs across 15 eukaryotic model organisms allowed us to identify distinct patterns that highlight the evolutionary trajectory of this protein family (Fig. 5c). Notably, no SPIN90-family proteins were identified in *Arabidopsis*, indicating a potential evolutionary loss or replacement by alternative functional analogs in plants. Intriguingly, however, SPIN90 orthologs in all examined metazoans contain an SDD, together with SH3 and armadillo repeat domains in a conserved N-terminal to C-terminal order with the exception of *Caenorhabditis* *elegans*, which lacks an SH3 domain. Furthermore, AlphaFold structural predictions of the SDD and armadillo repeat domains of these proteins indicate that these metazoan SPIN90 orthologs are likely to form dimers (Extended Data Fig. 10). Interestingly, even SPIN90 from the unicellular holozoan protist *Salpingoeca* *rosetta*, a choanoflagellate and the sister group of metazoans, also contains all three domains^44^. In contrast, two fungal species and other protists, such as *Tetrahymena thermophila* and *Dictyostelium discoideum* (slime mold) are predicted to contain armadillo repeat domains, within which the previously identified LRD is located but the SDD and SH3 domain are absent. This structural divergence reflects functional specifications aligned with the complexity of signaling networks in different evolutionary lineages. Indeed, the conservation of the three-domain and dimeric arrangement in choanoflagellates suggests that it is an ancient evolutionary adaptation specific to the holozoan clade, occurring before the emergence of multicellular animals^45^. This hints at an ancient evolutionary path that laid the foundation for the complex actin system observed in animals today. In the future, it will be important to explore the implications of SPIN90 dimerization in its regulation and cellular role in specifying bidirectional actin filament polymerization. This has the potential to affect the dynamics and geometry of the resulting actin networks in different cellular contexts.

## Methods

### Protein purification

Human SPIN90-C (residues 274–722) and its mutants were purified following the protocol described by Cao et al.^42^. Briefly, affinity chromatography was performed using a HisTrap HP column (Cytiva) followed by size exclusion using a Hiload Superdex 200 column (Cytiva). Human Arp2/3 complex (containing ArpC1B and ArpC5L) was purified following the protocol by Baldauf et al.^47^. Briefly, human Arp2/3 ArpC1B and ArpC5L subunits were cloned into a baculovirus vector, expressed in Sf21 insect cells (Life Technologies) and purified using a C-terminal Twin-Strep-tag on ArpC3 on a StrepTrap XT column (Cytiva) and subsequent Superdex 200 Increase 10/300 GL column (GE Healthcare). Mouse capping protein α1β2 was purified following the protocol described by Liu et al.^26^. Briefly, the steps were (1) coexpression of subunits in BL21 Star DE3 cells using a pRSFDuet-1 plasmid with an N-terminal 6×His tag fused to the α1 subunit; (2) affinity chromatography using Ni-NTA resin (Merck); (3) size-exclusion chromatography using Superdex 200 Increase 10/300 GL (GE Healthcare); and (4) ion-exchange chromatography using a HiTrap Q HP column (GE Healthcare). Some proteins used in that previous study were reused here^26^.

Mutant proteins were prepared using PCR-based site-directed mutagenesis using Q5 high-fidelity DNA polymerase (New England Biolabs, M0491) and the following primers: SPIN90 deletion mutant_Fwd, AGTTGGGCACTATATGAGGATGAGG; SPIN90 deletion mutant_Rev, CTTCCGGCGagCcAGGTCTG; SPIN90 glycine mutant_Fwd, cGGTGGTGGTGGtGATGAGGGTGTCATCCGCTGC; SPIN90 glycine mutant_Rev, CCACCaCCgCCACCGGcgTCGTCCTTCCGGCGag; Arp2/3 (C1BC5L) C1B mutant (F302A)_Fwd, GCTCGTGAACGGGCTCAAAATCTCG; Arp2/3 (C1BC5L) C1B mutant (F302A)_Rev, TGTAAGACCCCTTTGGCTTGATTGTTTCG; Arp2/3 (C1BC5L) C1B mutant (ΔAA298–308)_Fwd, TCGAGTGAAGGCGGTACTGCAGC; Arp2/3 (C1BC5L) C1B mutant (ΔAA298–308)_Rev, AGCTGTAAGACCCCTTTGGCTTG.

### Mass photometry

Purified recombinant SPIN90-C or its mutants was diluted to 30 nM with PBS. Large aggregates were eliminated by centrifuging at 21,130*g* for 10 min at 4 °C. Then, 2 µl of diluted protein was added to 18 µl of PBS in the well of a gasket on a TwoMP instrument (Refeyn) and events were recorded with AquireMP software (Refeyn) at room temperature. BSA (66 kDa; Thermo Fisher Scientific, 23209), ADH (150 kDa; Sigma-Aldrich, A7011) and urease (90, 272 and 544 kDa; Sigma-Aldrich, 94280) were used as standards.

### TIRF microscopy assays

Deep-ultraviolet-treated coverslips were passivated with mPEG silane overnight and thoroughly rinsed with ethanol and water. Flow chambers were prepared as described in Cao et al.^48^. G-actin (0.5 µM, 15% labeled with Alexa-488) was preincubated with SPIN90 and the Arp2/3 complex for 4 min in the incubation buffer containing 5 mM Tris-HCl pH 7.0, 50 mM KCl, 1 mM MgCl_2_, 0.2 mM EGTA, 0.2 mM ATP, 10 mM DTT and 1 mM DABCO at room temperature. Alternatively, 1 µM G-actin (15% labeled with Alexa-488) was incubated for over 10 min in the incubation buffer to generate spontaneously nucleated actin filaments.

Prepolymerized actin was then mixed with 0.5 µM G-actin (15% labeled with Alexa-568) in the imaging buffer, which included 0.1% BSA and 0.3% methylcellulose in addition to the incubation buffer, and loaded directly onto the TIRF microscope. As a control, incubation of 0.5 µM actin alone for 5 min resulted in fewer than five filaments per field of view, whereas incubation of 0.5 µM actin with 250 nM SPIN90 and 25 nM Arp2/3 produced more than 100 filaments per field of view. Image acquisition was performed at 25 °C. For each independent repeat, the fraction of filaments with double barbed ends was quantified. The mean and s.d. were then calculated and plotted.

Fiji software (version 2.14.0/1.54f) was used to analyze images manually^49^. To randomly select actin filaments for analysis, the red channel was turned off to avoid biasing filament selection according to their growth state. Prepolymerized filaments with strong 488-nm signals were excluded, as they were most likely actin bundles with uncontrollable numbers of filament ends. Prism 10 (10.1.1) software (GraphPad) was used to calculate all the statistical analysis.

### Cryo-EM sample preparation

Porcine βλ nonmuscle actin lyophilized powder (Hypermol, 8105-01) was dissolved in water to obtain a stock solution of 1 mg ml^−1^ (23.8 μM). To reconstitute SPIN90-C, the Arp2/3 complex and their nucleated actin filaments, reconstitution conditions were adapted from that used to generate actin branches with the exclusion of VCA and cortactin^26^. Specifically, 1.5 μM Arp2/3 complex, 14.3 μM SPIN90-C, 0.7 μM actin and 2.9 μM capping protein were mixed in 16.8 μl of reaction buffer (20 mM HEPES pH 7.5, 50 mM KCl, 1 mM EGTA, 1 mM MgCl_2_, 0.2 mM ATP and 1 mM DTT). The mixture was incubated at room temperature for 20 min. Then, 0.5 μl of 23.8 μM actin stock was added in nine steps. After each addition of actin, the mixture was incubated at room temperature for 20 min. Additionally, 0.6 μl of 80 μM capping protein was added together with the third and seventh additions of actin. At the end of the reaction, 100 μM phalloidin was added to stabilize the polymerized actin filaments.

Next, 4 μl of the mixture was applied to a glow-discharged C flat 1.2/1.3 Cu grid. The grid was plunge-frozen into liquid ethane using an EM GP2 Automatic Plunge Freezer (Leica). The sensor and backblotting parameters were as follows: additional movement of 0.3 mm, blotting time of 5 s, temperature of 25 °C and humidity of 95%.

### Cryo-EM data acquisition

Cryo-EM data were collected on a Titan KriosIV (Thermo Fisher Scientific) at the Diamond Electron Bioimaging Center (eBIC) equipped with a K3 detector and a BioQuantum energy filter (Gatan). The microscope was operated at an acceleration voltage of 300 kV with a nominal magnification of ×81,000 and a pixel size of 1.06 Å. A total of 12,512 videos were collected using EPU with the following parameters: super-resolution mode, a dose rate of 22.0 e^−^ per pixel per second, an exposure time of 2 s, 50 frames and a defocus range of −0.9 to −2.4 μm.

### Cryo-EM data processing

Cryo-EM data were processed using cryoSPARC (versions 3 and 4)^50^. Videos were motion-corrected using patch motion correction. Contrast transfer function (CTF) parameters were determined using patch CTF. A total of 10,235 micrographs with a CTF fit resolution better than 8 Å and total frame motion distance less than 45 pixels were selected for further data processing.

Initially, 2,138,010 particles were selected using blob picker, with a minimal particle diameter of 150 Å and maximum particle diameter of 200 Å. These particles were extracted with a binning factor of 4. Three rounds of 2D classification were used to remove obvious junk particles (for example, carbon and ice) and actin filament segments. Rather than relying on extensive 2D classification, the relatively inclusive set of remaining 87,216 particles were retained and subjected to ab initio reconstruction with three classes to pull out the SPIN90–Arp2/3-containing particles. A total of 13,727 unbinned particles from class 3 displaying the characteristic ArpC1B β propeller density were selected for homogeneous refinement, obtaining a reconstruction at 4.2 Å.

To further improve the resolution, the particles used in the initial reconstruction were subjected to another round of 2D classification to remove residual junk. The refined particle set was used for Topaz training. Using the obtained Topaz model, 26,606 and 39,254 particles were picked from the first and second halves of the dataset, respectively^51^. These two particle sets were subjected to ab initio reconstruction.

Particles from the class resembling the previous homogeneous refinement reconstruction were combined and subjected to multiple rounds of 2D classification to remove junk. Finally, the remaining 39,104 particles were refined using nonuniform refinement and the resolution was further improved after applying *C*_2_ symmetry. Global and local resolutions were estimated in cryoSPARC.

Particles from the final *C*_1_ reconstruction were used for 3D variability analysis in cryoSPARC with six modes to solve and a filter resolution of 8 Å (ref. ^52^). We evaluated the top three modes, with the top mode providing relevant structural flexibility insight while others showed minor variations; therefore, we used the top mode for subsequent analysis. The 3D variability result was displayed in intermediate mode with four frames, a var_range_percentile of 3%, a var_intermediate_width of 0 frame and a filtered resolution of 8 Å. Two reconstructions generated from the particle sets (frames 1 and 4) with the most divergent component values were used for model building.

To try to obtain the structure of SPIN90–Arp2/3 at the end of unidirectional actin filaments, 14,425 particles from 2D classes showing density at filament ends were selected and used to train Topaz for particle picking. Rare view particles were used for a further round of Topaz training and extraction. This approach yielded 23,637 particles (Extended Data Fig. 4c). However, subsequent 2D analysis revealed that (1) only a minority of particles showed evidence of SPIN90 density and (2) a severe preferred angular distribution prevented 3D reconstruction (Extended Data Fig. 4c).

### Model building

The Arp2/3 complex and four daughter filament subunits from Protein Data Bank (PDB) 8P94, along with the SPIN90 structure from PDB 6DEE, were rigidly fitted into one asymmetric unit of the cryo-EM map using ChimeraX (1.6.1), followed by molecular-dynamics-based flexible fitting using ISOLDE (1.6.0)^53,54^. Namdinator was used to optimize bond geometry^55^. Clashes, Ramachandran outliers and rotamer outliers were manually corrected using ISOLDE and Coot^56^. PHENIX (1.14) real-space refinement was used for refining the model^57^. Any residual clashes, Ramachandran outliers and rotamer outliers were again manually fixed using ISOLDE and Coot (0.8.9.1). The model was then duplicated and fitted into the density of the other asymmetric unit. Models used in 3D variability analysis in Supplementary Video 2 and Extended Data Fig. 3e were further refined from the above model using Namdinator and morphed using ChimeraX. For each model, the helical axis of nucleated actin filaments was measured in Pymol (2.5.4). The Cα displacement was displayed in ChimeraX using a custom script^58^.

### Phylogenetic analysis

Orthologs of SPIN90 in 15 model organisms were identified using InterPro (IPR030125) and Ensembl^59,60^. Using predictions from the AlphaFold database, the presence of the SH3 domain, SDD and armadillo repeat domains in these proteins was analyzed^61,62^. The dimeric structures of the representative SPIN90-family protein were predicted using the AlphaFold server^63^.

### Pyrene assay

To test the nucleation efficiency of Arp2/3 ± SPIN90, 8 nM Arp2/3 C1B/C5L complex, 400 nM SPIN90 and 2.5 µM G-actin (5% pyrene-labeled) were mixed and measured in a Safas Xenius fluorimeter at room temperature. The negative control consisted of the same amount of pyrene actin without SPIN90 and Arp2/3 complex. The competition assays were performed using varying concentrations of the SPIN90 mutant, in the presence of 400 nM WT SPIN90, 8 nM Arp2/3 C1B/C5L complex and 2.5 µM G-actin (5% pyrene-labeled). The experimental buffer contained 5 mM Tris-HCl (pH 7.0), 50 mM KCl, 1 mM MgCl_2_, 0.2 mM EGTA, 0.2 mM ATP, 10 mM DTT and 1 mM DABCO, all maintained at room temperature. For each experimental condition, the pyrene assay was repeated three times independently. The maximum polymerization rate for each pyrene curve was measured and plotted. The mean and s.d. for each condition were calculated. A two-tailed unpaired *t*-test was applied to analyze the statistical significance, with *P* values shown in the figures.

### Reporting summary

Further information on research design is available in the Nature Portfolio Reporting Summary linked to this article.

## Online content

Any methods, additional references, Nature Portfolio reporting summaries, source data, extended data, supplementary information, acknowledgements, peer review information; details of author contributions and competing interests; and statements of data and code availability are available at 10.1038/s41594-025-01665-8.

## Supplementary information

Reporting Summary

Supplementary Video 1 Cryo-EM reconstruction of SPIN90–Arp2/3 complex nucleated actin filaments.

Supplementary Video 2 A 3D variability analysis reveals the structural plasticity of SPIN90–Arp2/3 complex.

Supplementary Video 3 SPIN90 dimer interacts with a single Arp2/3 complex with two major binding sites.

Supplementary Video 4 SPIN90 binding promotes the conformational changes of Arp3 toward activation.

## Source data

Source Data Fig. 1 Statistical source data.

Source Data Fig. 3 Statistical source data.

Source Data Fig. 4 Statistical source data.

Source Data Extended Data Fig. 1 Unprocessed SPIN90 mutant protein purification gel.

Source Data Extended Data Fig. 3 Statistical source data.

Source Data Extended Data Fig. 7 Statistical source data.

## Data Availability

The cryo-EM reconstruction was deposited to the EM Data Bank under accession codes EMD-52580. The corresponding structural model was deposited to the PDB under the accession code 9I2B. Data and materials can be obtained from the corresponding authors upon request. Source data are provided with this paper.
