# Supplementary material for: Arp2/3-mediated bidirectional actin assembly by SPIN90 dimers
Source: Nat Struct Mol Biol. 2025 Sep 15;32(11):2262–71. doi: 10.1038/s41594-025-01665-8 (PMC12618257; doi:10.1038/s41594-025-01665-8)
Supplement: Supplementary file 1 — Reporting Summary [file 41594_2025_1665_MOESM1_ESM.pdf]

nature portfolio

Corresponding author(s): Way & Moores

Last updated by author(s): July 23 2025

Reporting Summary

Nature Portfolio wishes to improve the reproducibility of the work that we publish. This form provides structure for consistency and transparency in reporting. For further information on Nature Portfolio policies, see our Editorial Policies and the Editorial Policy Checklist.

Statistics

For all statistical analyses, confirm that the following items are present in the figure legend, table legend, main text, or Methods section.

n/a	Confirmed	
☐	☒	The exact sample size (*n*) for each experimental group/condition, given as a discrete number and unit of measurement
☐	☒	A statement on whether measurements were taken from distinct samples or whether the same sample was measured repeatedly
☐	☒	The statistical test(s) used AND whether they are one- or two-sided<br>*Only common tests should be described solely by name; describe more complex techniques in the Methods section.*
☒	☐	A description of all covariates tested
☒	☐	A description of any assumptions or corrections, such as tests of normality and adjustment for multiple comparisons
☐	☒	A full description of the statistical parameters including central tendency (e.g. means) or other basic estimates (e.g. regression coefficient) AND variation (e.g. standard deviation) or associated estimates of uncertainty (e.g. confidence intervals)
☐	☒	For null hypothesis testing, the test statistic (e.g. *F*, *t*, *r*) with confidence intervals, effect sizes, degrees of freedom and *P* value noted<br>*Give P values as exact values whenever suitable.*
☒	☐	For Bayesian analysis, information on the choice of priors and Markov chain Monte Carlo settings
☒	☐	For hierarchical and complex designs, identification of the appropriate level for tests and full reporting of outcomes
☒	☐	Estimates of effect sizes (e.g. Cohen's *d*, Pearson's *r*), indicating how they were calculated

Our web collection on statistics for biologists contains articles on many of the points above.

Software and code

Policy information about availability of computer code

Data collection	Cryo-EM data was collected using EPU software (Thermo Fisher Scientific) on eBIC Titan KriosIV (Thermo Fisher Scientific) equipped with a K3 detector and a BioQuantum energy filter (Gatan). Mass photometry data was collected using TwoMP instrument (Refeyn) and events were recorded with AquireMP software (Refeyn). The directional actin polymerisation assay was performed with TIRF microscopy (Nikon TiE inverted microscope, iLAS2, Gataca Systems) equipped with a 60× oil-immersion objective. Images were acquired using an Evolve EMCCD camera (Photometrics), controlled with the Metamorph software (version 7.10.4, from Molecular Devices). Pyrene assays were performed on a Safas Xenius fluorimeter, with 10 mm quartz glass cuvettes (00X105-10-40, from Hellma).
Data analysis	Cryo-EM data was processed using CryoSPARC v3 and v4.Model was built using ISOLDE(1.6.0),Namdinator,Coot(0.8.9.1) and Phenix(version 1.14). The helical axis of nucleated actin filaments is measured in Pymol (version 2.5.4). Structural figures and movies were made with ChimeraX(1.6.1). Fiji (version 2.14.0/1.54f) was used to analyse the TIRF data manually. Excel and Prism 10 (10.1.1) software (GraphPad) was used to calculate all the statistical analysis.

For manuscripts utilizing custom algorithms or software that are central to the research but not yet described in published literature, software must be made available to editors and reviewers. We strongly encourage code deposition in a community repository (e.g. GitHub). See the Nature Portfolio guidelines for submitting code & software for further information.

Data

Policy information about availability of data

All manuscripts must include a data availability statement. This statement should provide the following information, where applicable:

- Accession codes, unique identifiers, or web links for publicly available datasets
- A description of any restrictions on data availability
- For clinical datasets or third party data, please ensure that the statement adheres to our policy

The cryo-EM reconstruction is deposited in the Electron Microscopy Data Bank under the following accession codes: EMDB-52580. The corresponding structural model is deposited in the Worldwide Protein Data Bank under the accession code PDB ID: 9I2B.

Research involving human participants, their data, or biological material

Policy information about studies with human participants or human data. See also policy information about sex, gender (identity/presentation), and sexual orientation and race, ethnicity and racism.

Reporting on sex and gender	n/a
Reporting on race, ethnicity, or other socially relevant groupings	n/a
Population characteristics	n/a
Recruitment	n/a
Ethics oversight	n/a

Note that full information on the approval of the study protocol must also be provided in the manuscript.

Field-specific reporting

Please select the one below that is the best fit for your research. If you are not sure, read the appropriate sections before making your selection.

☒ Life sciences ☐ Behavioural & social sciences ☐ Ecological, evolutionary & environmental sciences

For a reference copy of the document with all sections, see nature.com/documents/nr-reporting-summary-flat.pdf

Life sciences study design

All studies must disclose on these points even when the disclosure is negative.

Sample size	The sample size for cryo-EM study was determined based on previous similar studies. Thousands of movies are needed to obtain a high resolution reconstruction. 12,512 movies were collected. After manually curate collected movies, 10,235 micrographs with CTF fit resolution < 8 Å and total full-frame motion distance < 45 pixels were selected for further data processing. For TIRF assays, for each condition and for each repeat, about 30 filaments were chosen randomly.
Data exclusions	Cryo-EM images were selected in a non-biased manner using well defined criteria (CTF fit resolution, total full-frame motion distance etc).
Replication	For the Cryo-EM dataset, similar images were obtained from 3 preliminary test dataset. Data for 3D reconstruction were collected from one grid. For biochemical assay, each experiments were repeated 2-3 times independently.
Randomization	During cryoEM data processing, each dataset was randomly split to two half for calculating gold-standard Fourier Shell Correlation (FSC).
Blinding	Blinding is not feasible for Cryo-EM study.

Reporting for specific materials, systems and methods

We require information from authors about some types of materials, experimental systems and methods used in many studies. Here, indicate whether each material, system or method listed is relevant to your study. If you are not sure if a list item applies to your research, read the appropriate section before selecting a response.

Materials & experimental systems

n/a	Involved in the study
☒	☐ Antibodies
☒	☐ Eukaryotic cell lines
☒	☐ Palaeontology and archaeology
☒	☐ Animals and other organisms
☒	☐ Clinical data
☒	☐ Dual use research of concern
☒	☐ Plants

Methods

n/a	Involved in the study
☒	☐ ChIP-seq
☒	☐ Flow cytometry
☒	☐ MRI-based neuroimaging

Plants

Seed stocks	n/a
Novel plant genotypes	n/a
Authentication	n/a
