# Supplementary figures and images for: Arp2/3-mediated bidirectional actin assembly by SPIN90 dimers
Source: Nat Struct Mol Biol. 2025 Sep 15;32(11):2262–71. doi: 10.1038/s41594-025-01665-8 (PMC12618257; doi:10.1038/s41594-025-01665-8)

SPIN90-C mutant  
 $\Delta 394-399$  G397-405

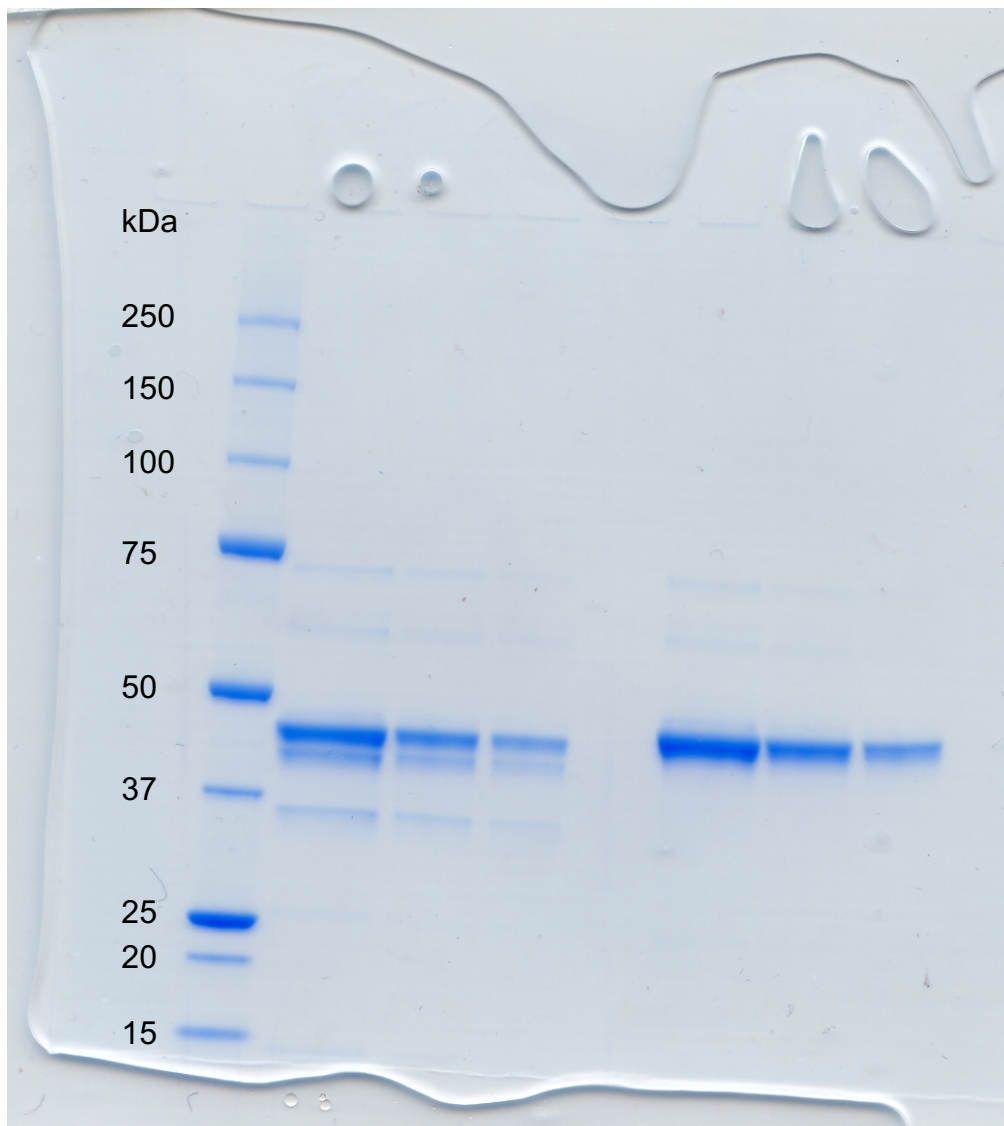

Supplement: Supplementary file 9 — Unprocessed SPIN90 mutant protein purification gel. [file 41594_2025_1665_MOESM9_ESM.pdf]
